# The psychological impact of Early Pregnancy Loss in Portugal: incidence and the effect on psychological morbidity

**DOI:** 10.3389/fpubh.2023.1188060

**Published:** 2023-06-22

**Authors:** Diana C. Gonçalves Mendes, Ana Fonseca, Mónica S. Cameirão

**Affiliations:** ^1^Faculdade de Ciências Exatas e da Engenharia & NOVA LINCS, Universidade da Madeira, Funchal, Portugal; ^2^Agência Regional para o Desenvolvimento de Investigação, Tecnologia e Inovação (ARDITI), Funchal, Portugal; ^3^University of Coimbra, Center for Research in Neuropsychology and Cognitive and Behavioral Intervention, Coimbra, Portugal

**Keywords:** Early Pregnancy Loss, miscarriage, perinatal grief, depression, anxiety, PTSD

## Abstract

**Introduction:**

Worldwide, up to a quarter of all recognized pregnancies result in Early Pregnancy Loss (EPL), also known as miscarriage. For many women, this is a traumatic experience that leads to persistent negative mental health responses. The most common morbidity reported in studies from different countries is complicated grief, usually comorbid with depression, anxiety, and Post-Traumatic Stress Disorder (PTSD). To our best knowledge, no studies characterizing the psychological impact of EPL have been made in Portugal.

**Methods:**

An online survey was conducted to evaluate clinical symptoms of perinatal grief, anxiety, depression, and PTSD in women who suffered a spontaneous loss within 20  weeks of gestation. Out of 1,015 women who answered this survey, 873 were considered eligible, and subsequently distributed in 7 groups according to the time passed between their loss and their participation in the study.

**Results:**

The proportion of women showing symptoms of all comorbidities was greater in those whose loss had happened within a month, and there was a significant gradual decrease over time in scores and proportions of clinical perinatal grief and PTSD. In terms of depression symptoms, scores dropped significantly in the group whose loss occurred 13–24  months before their participation but proportions oscillated without great changes in the other groups. Regarding anxiety, there were small oscillations, but there was no significant decrease of symptoms over time.

**Discussion:**

Overall, despite a general drop in scores for most morbidities over time, substantial proportions of women showed persistent symptoms of clinical morbidities 3  years or more after the loss. Therefore, it is essential to promote monitoring of possible complicated responses to the event, to provide appropriate and timely intervention to those women in need.

## 1. Introduction

In the last decades, several studies performed in different parts of the world have reported that 15 to 25% of all recognized pregnancies end up in Early Pregnancy Loss (EPL) (1). Further, the World Health Organization has reported that approximately one in every four confirmed pregnancies ends in loss before the 28th week of gestation (2). No consensus exists on whether losses are considered Early Pregnancy Losses before the 28, 24, or 20th week of gestation. However, here it will be defined as the fetal demise and removal from the womb at or before the 20th week of gestation (1, 3). Knowing the actual incidence is challenging, as many early losses are not accounted for. In the particular case of Portugal, the National Statistics Institute (INE) only classifies fetal deaths as before or after 32 weeks of gestation. Even though 40% of fetal deaths correspond to losses at a gestational age of 32 weeks or less (4, 5), it is unclear how many correspond to losses at or before the 20th week of gestation.

The physical risks to the mother’s well-being are low in cases of EPL if appropriately managed (6). Nevertheless, an invasive medical procedure is required in some cases, which could be perceived as physical trauma (6, 7). In a study performed by Beutel et al. (8), 48% of the women participating had no significant emotional reactions the first days after the loss, but more recent studies support that a significant number of women end up experiencing very sad feelings after pregnancy loss in general, and for some, these emotions can be very intense (3, 9, 10). Feelings of shock, stress, guilt, anger, distress, confusion, sleep and eating disturbances, loneliness, low self-esteem, hopelessness, and helplessness are among the most commonly reported (11). Several reviews and/or meta-analytic studies have shown that, while many women who suffered an EPL cope with their loss naturally and without complications, it can lead to negative mental health responses in about 25–50% of this population (6, 12, 13).

Grief is a normal response to the loss of a significant one, and it is characterized by feelings of sadness, sleep and eating disturbances, loneliness, longing, anger, and thoughts, memories, and images of the deceased (14). We usually cope naturally and adapt to the world without our loved one, but when grief disrupts the person’s daily functioning, and causes social impairment, it is considered Complicated Grief (CG) (15). Previous studies suggest that high levels of grief remain the first few days or weeks after an EPL, but tend to reduce gradually after the 4 or 7th week, and may resolve about 3 or 4 months after the event (Lee and Slade, 1996) (16–18). However, other studies have shown that, despite a typical decline 6 months after the loss, intense feelings of grief can persist 2 years or more (19, 20). A population studied by Krosch et al. (21) with a mean time after the loss of 4 years, showed a proportion of 57% with clinically significant levels of perinatal grief; and a later study by deMontigny et al. (22), which compared mean scores of perinatal grief in women whose miscarriage had occurred 0–6 months, 7–12 months, 1–2 years, and over 2 years before, reported that the scores of perinatal grief did not vary significantly over time.

After an EPL, grieving women tend to show high levels of other comorbidities, the most reported being depression, anxiety, and post-traumatic stress disorder (PTSD) symptoms (22–26). Clinical depression and anxiety, commonly comorbid and thus often mentioned together, are likely to have their first episode triggered by a psychosocial stressor, such as what can be experienced with an Early Pregnancy Loss (27). Some studies have reported short-term anxiety to affect approximately 20–40%, and depression 15–55% of women who have suffered an EPL (28, 29). A more recent study showed even greater morbidity, with 73% of their sample presenting symptoms of depression, and 100% with moderate symptoms of anxiety (30). These studies seem to support some early studies reporting that anxiety might be more frequent and have higher morbidity than depression in this population (18, 31). However, another study reported that, while 10.5% of their sample showed symptoms of depression, 6.8% showed symptoms of anxiety (32). Furthermore, a number of studies have shown that depression and anxiety symptoms can prevail, not only in the immediate weeks after the loss but as far as 6 months, 1, or even 3 years after an EPL.

Focusing on anxiety, some studies have shown that anxiety levels decreased over time, more specifically within 4 weeks and 9 months after the event (18, 24, 25). Nevertheless, other studies have reported no significant changes in the frequency or mean scores of anxiety symptoms within a week and 2–5 years after the loss (22, 33, 34).

Regarding depression, studies have shown that 16–22% of women present symptoms of clinical depression within the first month post-loss, which are significantly reduced 3–4 months after the loss (18, 24, 34, 35). Some studies have investigated the incidence of depression symptoms covering even longer timeframes. An early study covering a timeline from 2 weeks to 6 months after the loss, reported that about 36% of their participants showed moderate-to-severe depression symptoms 2 weeks after the loss, a proportion that gradually decreased but remained relevant 6 months after the loss, with almost 11% of the latter experiencing major depression (36). A relatively recent study covering a slightly longer timeframe reported more modest frequencies, with 10% of their sample showing symptoms of moderate/severe depression at 1 month, and presenting a small decline to 7% at the 9-month mark (25). Other studies have focused on emphasizing the reduction of depression symptoms over time, reflecting on the mean scores over time shown in their samples rather than the frequency of clinically significant cases. The study of deMontigny et al. (22) showed that women who experienced EPL within the past 6 months had higher scores of depressive symptoms than those who experienced it between 7 and 12 months, 1–2 years, or over 2 years ago. Further, an earlier study performed by Broen et al. (33) extended their time frame to the span of 5 years and reported a decrease in depression scores when compared to 10 days after the loss. Interestingly, despite not reaching scores or proportions as high as those seen in the first 3–6 months post-loss, some studies have reportedly shown a slight spike or increase in depression symptoms 1–2 years after the loss, disrupting the generally gradual decline (22, 37).

Finally, symptoms of Post-Traumatic Stress Disorder (PTSD) are also often observed in women that underwent an EPL. PTSD can be defined as a trauma resulting from exposure to a threatening event that causes profound discomfort, and it is characterized by avoidance, numbing, the presence of intrusive thoughts, hyperarousal, and hypervigilance for an imminent traumatic event (38, 39). For women that undergo EPL, hospitalization, pain, the sole sight of blood, fetal tissue, the fetus, or even the subjective perception that a baby is dying inside the womb can be traumatic enough for the appearance of PTSD symptoms (24, 40). Recent studies suggest that around 10–45% of these women show symptoms of post-traumatic stress disorder (39, 41). Regarding its persistence in time, an early study by Engelhard et al. (42), reported that symptoms of PTSD were visible in 25% of their sample at the 1-month mark, and had substantially decreased by the 4-month mark. Further studies have indicated the persistence of PTSD symptoms at 1 month, 9 months, and even years after the loss, and others have brought evidence of a gradual decline of PTSD symptoms 1–9 months after the event (21, 24, 25).

The psychological effects beforehand described are sometimes overlooked by overexposed clinicians, which has led, in some cases, to negative outcomes in terms of quality of life, physical health, ability to work, depression and anxiety in subsequent pregnancies, among others (22, 24, 43). For instance, EPL has been linked with an increased risk of avoidant behaviors (e.g., alcohol and drug dependence), sexual dysfunction, and couple separation (8, 40, 44). Hence, characterizing or having actual data on the impact of EPL on the mental health of women in different nations and cultures is key to understanding and highlighting the need for preventive interventions. To our best knowledge, no extensive studies on the psychological impact of EPL have been conducted in Portugal. In fact, the research found has been mostly focused on the parents’ experience and feelings faced after the loss [e.g. (45, 46)]. Such studies have undoubtedly provided essential information to understand these women’s emotional experiences, but generally provide no rigorous data regarding the actual proportions of these women that might be in need of help in terms of complicated psychological responses. Aiming to address these issues, we conducted an online survey to evaluate the psychological impact of EPL on women residing in Portugal, in terms of clinical symptoms of perinatal grief, depression, anxiety, and PTSD. In addition, we aimed at assessing how the intensity and persistence of these symptoms was modulated by the time passed since the loss to determine the influence of time on possible symptoms of clinical morbidities.

## 2. Materials and methods

### 2.1. Design and procedures

This was a descriptive cross-sectional research where we characterized the target population and compared the variables at a single point in time. This study had four dependent variables: the scores of depression, anxiety, perinatal grief, and PTSD symptoms. Our independent variable was the time passed since the miscarriage event, and it had seven levels according to the months passed since the loss: 0–1, 2–3, 4–6, 7–12, 13–24, 25–36, and 37 or more. Other variables were not considered for statistical analysis because they were collected for descriptive purposes only. We developed an online survey with a self-reporting questionnaire using Google Forms. The questionnaire was disseminated through ads on social networks, associations for support regarding fertility and pregnancy loss, and posters sent to the main Gynecology and Obstetrics units in the country for display. Data collection started on February 23, 2022 and ended on July 4, 2022. The participants were informed of the objectives of the study, the data collection procedure, and the tasks to complete. Further, we ensured the protection of their data and highlighted their right to quit the study at any time. Considering the online nature of the questionnaire, consent to participate in the study was obtained by ticking a checkbox. Only after this verification, the participants had access to the questionnaire. Whoever decided not to participate was taken to a check-out section. This study was approved by the Data Protection Committee (February 9, 2022) and the Ethics Committee (February 17, 2022—Number P27) of the University of Madeira.

### 2.2. Sample

This was a self-selecting sample of adult women who have suffered a pregnancy loss and were eligible to fill out the questionnaire if they met the following inclusion criteria: (1) Having suffered a pregnancy loss in the first 20 weeks of gestation, (2) Being ≥18 years old, and (3) Being a resident in Portugal. Participants were not eligible if they had (1) termination for medical reasons or (2) voluntary abortion. The survey was answered by 1,015 women. After the survey was closed, a close analysis of the responses obtained was performed. Out of the 1,015 respondents, 142 were excluded (8 for not being Portugal residents; 14 for suffering a pregnancy loss over the 20-week gestational age mark; 4 for carrying out a voluntary interruption of pregnancy; 6 for not providing key information or having faulty data; and 110 for undergoing a medical interruption of pregnancy), leaving a sample of 873 eligible responses. The 873 eligible responses were distributed as follows: Group 0–1, *n* = 131; Group 2–3, *n* = 78; Group 4–6, *n* = 146; Group 7–12, *n* = 107; Group 13–24, *n* = 135; Group 25–36, *n* = 65; and Group 37+, *n* = 211.

### 2.3. Materials

The online survey comprises clinically validated questionnaires and questions based on prior surveys in the field (11, 22). The survey was created under the expert judgment of co-authors with experience in perinatal mental health, who ensured content validity of the survey. The survey was composed of the following sections:

#### 2.3.1. Socio-demographic and clinical data

Participants were asked for socio-demographic information such as their age, nationality, place of residence, profession, and others. It was also requested basic clinical information about previous pregnancies, fertility problems, diagnosed mental health issues, and any previous or recurring treatments. It was aimed at characterizing this population and identifying factors that could potentially influence the psychological impact of pregnancy loss.

#### 2.3.2. The hospital anxiety and depression scale

It is a screening device for anxiety and depression that, despite its name, has proven to be valid in primary care and useful in psychiatric and psychological work (47, 48). Different studies have shown its internal consistency and satisfactory validity for populations of different ages and gender (49, 50). Its Portuguese translation by Pais-Ribeiro et al. has shown similar properties to the original and confirmed it as reliable and valid (47*). This instrument is used in hospital and clinical environments to measure levels of anxiety and depression symptoms using two scales: seven items to measure depression, and other seven items to measure anxiety. These are to be answered using a Likert scale of four points (0–3). After summing up all the items of each subscale, their score ranges from 0 to 21, being 0–7 considered normal, while 8–10 indicates symptoms of mild, 11–14 moderate, and 15–21 severe anxiety or depression (47).

#### 2.3.3. Posttraumatic stress disorder checklist for DSM-5

This instrument has shown to be valid and reliable in both its initial evaluation and in more recent studies (53*, 51, 52), and it is the instrument of choice by the PTSD: National Center for PTSD of the U.S. Department of Veterans Affairs (53*). Its Portuguese version has also shown validity and reliability in assessing PTSD symptomatology (54). It evaluates post-traumatic stress symptoms, and is composed of 20 items corresponding to post-traumatic stress symptoms identified by the Diagnostic Statistical Manual of Mental Disorders—5th Edition (DSM-5). Respondents answer to what extent they were affected by each symptom the previous month on a five-point Likert scale (0–4). There are several rules for provisional diagnosis using this tool, but the National Center for PTSD indicates that a provisional diagnosis can be determined by: (1) summing all 20 items and using a cut-off score of 31–33; (2) a single item considered symptomatic if rated equal or above the cutoff point of 2 (moderately). Here we use the total score, with a score of 33 or higher indicating probable PTSD and that the participant may benefit from PTSD follow-up, while less than 33 indicates that the symptoms are subthreshold or do not meet the criteria for PTSD (55).

#### 2.3.4. Perinatal grief scale—reduced version

This instrument has been validated worldwide for different types of pregnancy losses, showing high internal consistency (56–58). Its Portuguese version has also been validated with good levels of reliability (57). It evaluates the level of grief resolution after a pregnancy loss, fetal death, neonatal death, or ectopic pregnancy. It is composed of 33 items measured on a scale of five points (1–5). The final score is obtained by summing all the elements (with two reversed questions) and can vary from 33 to 165. It can be interpreted in different ways, but the general interpretation uses a threshold of 91 points, with a score of 91 or above considered to represent potential psychiatric morbidity (58).

#### 2.3.5. Information about the loss

Participants were asked about their last pregnancy loss and the support received from their social network during the pregnancy and the loss process. It was aimed at registering relevant data about the loss such as rituals performed, the communication with their social environment, and the impact of the loss on their marital relationships, among others, to identify factors that could influence the psychological impact of the loss. The results of this section that are not related to the time passed since the loss are out of the scope of this paper.

#### 2.3.6. Mental health responses in the context of the loss

This section addressed satisfaction with the hospital environment, the health practitioners’ behavior, the information provided regarding psychological support after the loss, and others. The goal was to characterize the participants’ feelings and thoughts on their hospital experience at the moment of the loss. The results of this section are out of the scope of this paper.

#### 2.3.7. Lifestyle and leisure profile

Participants answered questions regarding the frequency they exercised, played sports, and other activities they perform to relax. It was aimed at recording the participants’ preferences regarding leisure and distraction activities, which indicates the return to normality that is intended to be reached during the grief process. The results of this section are out of the scope of this paper.

The participants were informed that filling out this questionnaire would take approximately 20 min.

### 2.4. Data analysis

Categorical variables are presented as frequencies and/or percentages, while quantitative data are presented through their mean ± standard deviation. To carry out the analysis of the psychological impact the event had on the target population and its frequency in time after the event, the 873 eligible responses were distributed into seven groups based on the time that had passed between the EPL and their participation in the study: Group 0–1 (1 month or less); Group 2–3 (2–3 months); Group 4–6 (4–6 months); Group 7–12 (7 months to 1 year); Group 13–24 (1–2 years); Group 25–36 (2–3 years); and Group 37+ (over 3 years). One-way independent samples ANOVA tests were used to measure the effect of time after the loss on the scores obtained from the different scales, using time after the loss as the independent variable with seven levels (corresponding to the seven groups). Effect sizes are reported using eta squared. *Post hoc* multiple comparisons were performed, with a Bonferroni correction, to determine significant changes between time groups. When reporting the proportions of participants with above-threshold symptoms in the different dependent variables, the Pearson’s Chi-square (χ^2^) test was used to test for significance across proportions. All statistical analyses were done using SPSS (59) and the threshold for significance was set at 5% (α = 0.05).

## 3. Results

### 3.1. Socio-demographic and clinical characterization

Regarding the socio-demographic data collected, the age of the participants ranged from 21 to 57 years (*M* = 36.04, SD = 4.9), 99% (*n* = 864) of the sample was white/Portuguese white/of European origin, 99% (*n* = 864) of Portuguese nationality, and 98.6% (*n* = 861) non-migrants (Table 1). All the districts and autonomous regions of Portugal were represented. A majority of 88.9% (*n* = 776) were married or in a civil union/domestic partnership and 8.8% (*n* = 77) were single. 61.7% (*n* = 539) had living children. 80.2% (*n* = 700) had higher education, 93.2% (*n* = 814) were employed, and 96.6% (*n* = 843) earned more than the minimum wage.

**Table 1:** Socio-demographic and clinical data per time group, with the highest values in bold.

		Time since the loss						
		0–1	2–3	4–6	7–12	13–24	25–36	37+
Age		M = 34.89 (SD = 4.9)	M = 34.46 (SD = 4.5)	M = 33.75 (SD = 4.9)	M = 35.01 (SD = 4.8)	M = 36.32 (SD = 4.3)	M = 36.83 (SD = 4.6)	M = 39.02 (SD = 4.1)
Nationality	Portuguese	**98.5% (*n* = 129)**	**100% (*n* = 78)**	**100% (*n* = 146)**	**97.2% (*n* = 104)**	**99.3% (*n* = 134)**	**100% (*n* = 65)**	**98.6% (*n* = 208)**
	Others	1.5% (*n* = 2)	__	__	2.7% (*n* = 3)	0.7% (*n* = 1)	__	10.0% (*n* = 2)
Ethnicity	White/Portuguese	**97.7% (*n* = 128)**	**100% (*n* = 78)**	**100% (*n* = 146)**	**96.3% (*n* = 103)**	**98.5% (*n* = 133)**	**100% (*n* = 65)**	**100% (*n* = 211)**
	White/European origin							
	Black/Portuguese black/African descendant/African origin	2.3% (*n* = 3)	__	__	1.9% (*n* = 2)	1.5% (*n* = 2)	__	__
	Asian/Portuguese Asian/Asian origin	__	__	__	__	__	__	__
	Gypsy/Portuguese gypsy	__	__	__	__	__	__	__
	Other	__	__	__	1.9% (*n* = 2)	__	__	__
Immigration Status	Immigrant	3.8% (*n* = 5)	__	__	2.8% (*n* = 3)	1.5% (*n* = 2)	__	0.9% (*n* = 2)
	Non-immigrant	**96.2% (*n* = 126)**	**100% (*n* = 78)**	**100% (*n* = 146)**	**97.2% (*n* = 104)**	**98.5% (*n* = 133)**	**100% (*n* = 65)**	**99.1% (*n* = 209)**
Marital Status	Married/Civil union	**86.3% (*n* = 113)**	**89.7% (*n* = 70)**	**90.4% (*n* = 132)**	**84.1% (*n* = 90)**	**91.1% (*n* = 123)**	**87.7% (*n* = 57)**	**90.5% (*n* = 191)**
	Divorced	2.3% (*n* = 3)	2.6% (*n* = 2)	0.7% (*n* = 1)	2.8% (*n* = 3)	__	1.5% (*n* = 1)	3.3% (*n* = 7)
	Single	11.5 (*n* = 15)	7.7% (*n* = 6)	8.9% (*n* = 13)	13.1% (*n* = 14)	8.9% (*n* = 12)	9.2% (*n* = 6)	5.2% (*n* = 11)
	Widow	__	__	__	__	__	1.5% (*n* = 1)	0.9% (*n* = 2)
Schooling	Primary education (Year 4)	__	__	__	__	__	__	__
	Primary education (Year 6)	__	__	__	__	__	__	__
	Lower secondary education (Year 9)	0.8% (*n* = 1)	2.6% (*n* = 2)	1.4% (*n* = 2)	1.9% (*n* = 2)	0.7% (*n* = 1)	__	0.9% (*n* = 2)
	Upper-secondary education	16.0% (*n* = 21)	14.1% (*n* = 11)	8.2% (*n* = 12)	13.1% (*n* = 14)	8.9% (*n* = 12)	6.2% (*n* = 4)	7.1% (*n* = 15)
	Vocational education	6.1% (*n* = 8)	1.3% (*n* = 1)	6.2% (*n* = 9)	11.2% (*n* = 12)	6.7% (*n* = 9)	3.1% (*n* = 2)	3.3% (*n* = 7)
	Higher education	**76.5% (*n* = 100)**	**82.1% (*n* = 64)**	**83.6% (*n* = 122)**	**72.8% (*n* = 78)**	**83.7% (*n* = 113)**	**90.8% (*n* = 59)**	**88.6% (*n* = 187)**
	Other	0.8% (*n* = 1)	__	0.7% (*n* = 1)	0.9% (*n* = 1)	__	__	__
Employment Status	Employed	**96.2% (*n* = 126)**	**94.9% (*n* = 74)**	**93.8% (*n* = 137)**	**94.4% (*n* = 101)**	**93.3% (*n* = 126)**	**87.7% (*n* = 57)**	**91.5 (*n* = 193)**
	Unemployed	3.8% (*n* = 5)	5.1% (*n* = 4)	6.2% (*n* = 9)	5.6% (*n* = 6)	6.7% (*n* = 9)	12.3% (*n* = 8)	8.5% (*n* = 18)
Wage	More than 705 euros	**93.9% (*n* = 123)**	**97.4% (*n* = 76)**	**97.3% (*n* = 142)**	**97.2% (*n* = 104)**	**97.8% (*n* = 132)**	**95.4% (*n* = 62)**	**96.7% (*n* = 204)**
	Less than 705 euros	6.1% (*n* = 8)	2.6% (*n* = 2)	2.7% (*n* = 4)	2.8% (*n* = 3)	2.2 (*n* = 3)	4.6% (*n* = 3)	3.3% (*n* = 7)
Living children	With living children	41.2% (*n* = 54)	44.9% (*n* = 35)	35.6% (*n* = 52)	**57.0% (*n* = 61)**	**70.4% (*n* = 95)**	**75.4% (*n* = 49)**	**91.5% (*n* = 193)**
	Without living children	**58.8% (*n* = 77)**	**55.1% (*n* = 43)**	**64.4% (*n* = 94)**	43.0% (*n* = 46)	29.6% (*n* = 40)	24.6% (*n* = 16)	8.5% (*n* = 18)
Number of pregnancy losses	One only	**69.5% (*n* = 91)**	**70.5% (*n* = 55)**	**78.1% (*n* = 114)**	**69.2% (*n* = 74)**	**56% (*n* = 76)**	**64.6% (*n* = 42)**	**72.0% (*n* = 152)**
	Two or more	30.5% (*n* = 40)	29.5% (*n* = 23)	21.9% (*n* = 32)	30.8% (*n* = 33)	43.7% (*n* = 59)	35.4% (*n* = 23)	28.0% (*n* = 59)
Pregnant at the moment	Yes	3.8% (*n* = 5)	14.1% (*n* = 11)	30.8% (*n* = 45)	**50.5% (*n* = 54)**	17.8% (*n* = 24)	13.8% (*n* = 9)	7.6% (*n* = 16)
	No	**96.2% (*n* = 126)**	**85.9% (*n* = 67)**	**69.2% (*n* = 101)**	49.5% (*n* = 53)	**82.2% (*n* = 111)**	**86.2% (*n* = 56)**	**92.4% (*n* = 195)**
Previous infertility diagnosis	Yes	18.3% (*n* = 24)	20.5% (*n* = 16)	11.6% (*n* = 17)	8.4% (*n* = 9)	20.7% (*n* = 28)	24.6% (*n* = 16)	12.3% (*n* = 26)
	No	**81.7% (*n* = 107)**	**79.5% (*n* = 62)**	**88.4% (*n* = 129)**	**91.6% (*n* = 98)**	**79.3% (*n* = 107)**	**75.4% (*n* = 49)**	**87.7% (*n* = 185)**
Has resorted to MAR techniques	Yes	14.5% (*n* = 19)	17.9% (*n* = 14)	13.0% (*n* = 19)	8.4% (*n* = 9)	17.8% (*n* = 24)	18.5% (*n* = 12)	10.0% (*n* = 21)
	No	**85.5% (*n* = 112)**	**82.1% (*n* = 64)**	**87.0% (*n* = 127)**	**91.6% (*n* = 98)**	**82.2% (*n* = 111)**	**81.5% (*n* = 53)**	**90% (*n* = 190)**
Previous mental health complication diagnosis	Yes	25.2% (*n* = 33)	20.5% (*n* = 16)	29.5% (*n* = 43)	27.1% (*n* = 29)	34.1% (*n* = 46)	29.2% (*n* = 19)	30.3% (*n* = 64)
	No	**74.8% (*n* = 98)**	**79.5% (*n* = 62)**	**70.5% (*n* = 103)**	**72.9% (*n* = 78)**	**65.9% (*n* = 89)**	**70.8% (*n* = 46)**	**69.7% (*n* = 147)**
Mental health treatment received	Received in the past and currently receiving	12.2% (*n* = 16)	19.2% (*n* = 10.3)	17.8% (*n* = 26)	13.1% (*n* = 14)	12.6% (*n* = 17)	15.4% (*n* = 10)	17.5% (*n* = 37)
	Received in the past but no longer receiving	24.4% (*n* = 32)	10.3% (*n* = 8)	21.2% (*n* = 31)	21.5% (*n* = 23)	26.7% (*n* = 36)	24.6% (*n* = 16)	22.3% (*n* = 47)
	Never	**63.4% (*n* = 83)**	**70.5% (*n* = 55)**	**61% (*n* = 89)**	**65.4% (*n* = 70)**	**60.7% (*n* = 82)**	**60% (*n* = 39)**	**60.2% (*n* = 127)**

Data collected on some basic clinical and mental health characteristics showed that most of our sample (69.2%, *n* = 604) reported to have had only one loss up until their participation in this study. 84.4% (*n* = 737) had never been diagnosed with any infertility issues, and a majority of 86.5% (755) of the sample had never gone through any Medically Assisted Reproduction (MAR) technique to get pregnant. The self-reported mental health history collected showed that 71.4% (*n* = 623) had not been diagnosed with any mental health issue (e.g., anxiety or depression) by the time of their participation in this study, and that 62.4% (*n* = 545) had not received any mental health treatment neither before or at the moment of participating in the study (Table 1).

### 3.2. Information regarding the loss

The participants were asked to provide information about their last instance of an EPL. The information reported by the participants showed that the lost pregnancy had been planned for 81.4% (*n* = 711) of our sample and it had been spontaneous (without recurring to MAR) for 91.1% (*n* = 795). The loss was of a one-embryo/fetus pregnancy for 95.6% (*n* = 835) of our sample. Finally, 77.5 (*n* = 677) of our sample reported the clinical cause of their loss to be unknown (Table 2).

**Table 2:** Self-reported information about the participants’ last Early Pregnancy Loss instance per time group, with the highest values in bold.

		Time since the loss						
		0–1	2–3	4–6	7–12	13–24	25–36	37+
Planned pregnancy	Yes	**86.3% (*n* = 113)**	**85.9% (*n* = 67)**	**82.9% (*n* = 121)**	**81.3% (*n* = 87)**	**81.5% (*n* = 110)**	**73.8% (*n* = 48)**	**78.2% (*n* = 165)**
	No	13.7% (*n* = 18)	14.1% (*n* = 11)	17.1% (*n* = X)	18.7% (*n* = 20)	18.5% (*n* = 25)	26.2% (*n* = 17)	21.8% (*n* = 46)
Pregnancy process	MAR	11.5% (*n* = 15)	11.5% (*n* = 9)	9.6% (*n* = 14)	9.3% (*n* = 10)	9.6% (*n* = 13)	12.3% (*n* = 8)	4.3% (*n* = 9)
	Spontaneous	**88.5% (*n* = 116)**	**88.5% (*n* = 69)**	**90.4% (*n* = 132)**	**90.7% (*n* = 97)**	**90.4% (*n* = 122)**	**87.7% (*n* = 57)**	**95.7% (*n* = 202)**
Type of pregnancy	One embryo/Fetus	**97.7% (*n* = 128)**	**96.2% (*n* = 75)**	**97.9% (*n* = 143)**	**92.5% (*n* = 99)**	**97% (*n* = 131)**	**89.2% (*n* = 58)**	**95.3% (*n* = 201)**
	Twins	2.3% (*n* = 3)	3.8% (*n* = 3)	2.1% (*n* = 3)	7.5% (*n* = 8)	3% (*n* = 4)	10.8% (*n* = 7)	4.7% (*n* = 10)
Clinical cause of the loss	Unknown	**70.2% (*n* = 92)**	**79.5% (*n* = 62)**	**79.5% (*n* = 116)**	**79.4% (*n* = 85)**	**77% (*n* = 104)**	**81.5% (*n* = 53)**	**78.2% (*n* = 165)**
	Congenital and chromosomal anomalies	8.4% (*n* = 11)	6.4% (*n* = 5)	5.5% (*n* = 10)	5.5% (*n* = 6)	2.9% (*n* = 4)	3.1% (*n* = 2)	5.2% (*n* = 11)
	Maternal health problems	0.8% (*n* = 1)	__	0.7% (*n* = 1)	0.9% (*n* = 1)	1.5% (*n* = 2)	__	1.9% (*n* = 4)
	Pregnancy complications	__	__	0.7% (*n* = 1)	0.9% (*n* = 1)	__	__	__
	Fetal-fetus transfusion syndrome	__	__	__	__	__	3.1% (*n* = 2)	10% (*n* = 2)
	Ectopic pregnancy	6.1% (*n* = 8)	5.1% (*n* = 4)	4.8% (*n* = 7)	3.7% (*n* = 4)	5.9% (*n* = 8)	6.2% (*n* = 4)	4.7% (*n* = 10)
	Other	14.9% (*n* = 19)	9.1% (*n* = 7)	9.1% (*n* = 13)	10% (*n* = 11)	12% (*n* = 17)	9% (*n* = 6)	9.8% (*n* = 20)

### 3.3. Perinatal grief

The mean scores of perinatal grief were the highest in the group whose loss happened within a month. There was a significant effect of [*F*(6, 866) = 7.67, *p* < 0.001]. The effect size was small-to-medium, η^2^ = 0.051. Multiple comparisons with the 0–1 months post-loss group indicated that the mean scores decreased significantly after the 4–6-month mark (Table 3).

**Table 3:** Mean scores per time group in the PGS, HADS Anxiety Subscale, HADS Depression Subscale, and the PCL-5 and significance.

	Time since the loss							
	0–1	2–3	4–6	7–12	13–24	25–36	37+	*p* value^1^
PGS	**M = 89.84 (SD = 24.39)**	**M = 79.63 (SD = 26.72)**	**M = 80.18 (SD = 26.13)** ^*^	**M = 77.90 (SD = 27.51)** ^*^	**M = 76.32 (SD = 24.13)** ^**^	**M = 70.38 (SD = 25.00)** ^**^	**M = 72.66 (SD = 24.21)** ^**^	**<0.001**
HADS anxiety	M = 8.98 (SD = 3.68)	M = 7.73 (SD = 4.15)	M = 8.45 (SD = 4.40)	M = 9.13 (SD = 4.30)	M = 7.73 (SD = 4.10)	M = 8.05 (SD = 4.36)	M = 8.42 (SD = 4.03)	0.061
HADS depression	**M = 6.42 (SD = 3.50)**	**M = 5.06 (SD = 3.88)**	**M = 5.73 (SD = 3.90)**	**M = 5.55 (SD = 3.80)**	**M = 4.82 (SD = 3.31)** ^*^	**M = 4.83 (SD = 3.55)**	**M = 5.69 (SD = 3.94)**	**0.011**
PCL-5	**M = 24.37 (SD = 13.68)**	**M = 20.36 (SD = 14.12)**	**M = 23.29 (SD = 15.42)**	**M = 21.49 (SD = 15.35)**	**M = 18.84 (SD = 13.90)** ^*^	**M = 16.92 (SD = 14.47)** ^*^	**M = 18.41 (SD = 12.99)** ^*^	**<0.001**

1 *p* value results of one-way ANOVA tests.

* Significance of a value of *p* between 0.001 and 0.05 in *post hoc* comparisons to baseline (0–1).

** Significance of a value of *p* < 0.001 in *post hoc* comparisons to baseline (0–1).

**Figure 1:**
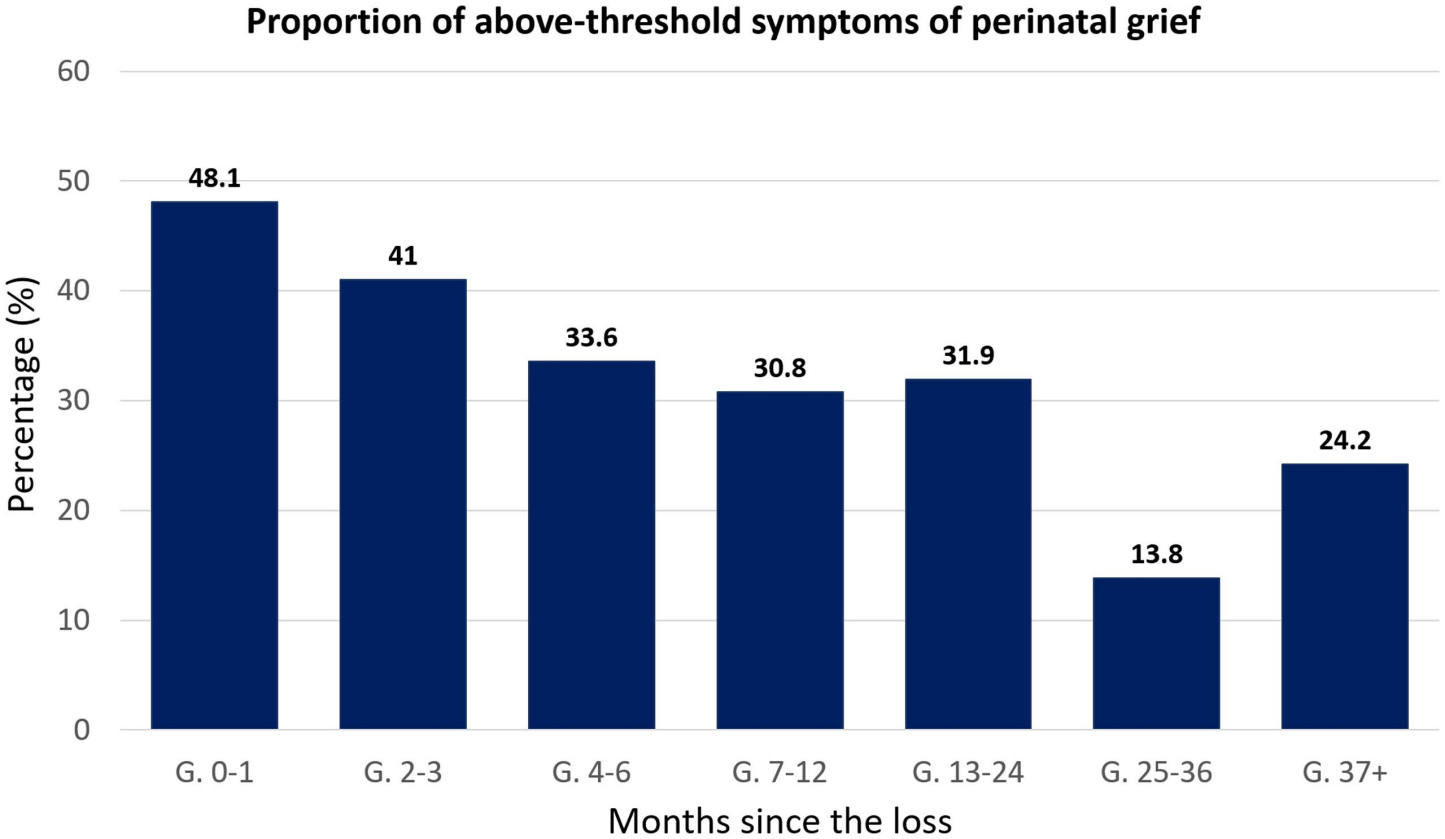
Bar chart representing the percentage of individuals showing above-threshold symptoms of perinatal grief, categorized according to the time passed between the loss and their participation in the study.

We compared the different groups regarding the frequency that probable clinical levels of perinatal grief were found. A cut-off score of 91 was used to define whether the sample showed probable clinical levels of perinatal grief (58) (Figure 1). A chi-square (χ^2^) analysis of the proportions of above-threshold Perinatal Grief across groups showed a significant difference, χ^2^ (6, *N* = 873) = 34.5, *p* < 0.001. The effect size was medium, with 𝝓 = 0.20. We observed greater proportions of clinical symptoms in the 0–1-month group that gradually decreased in the older groups, with a greater decrease after 4–6 months after the loss (Figure 1). From this point on, the frequency of clinical symptoms did not change much until a considerable drop was observed in the 25–36 months post-loss group. However, the decline in proportions did not continue in the 37+ months post-loss group, which showed a nearly 10% increase when compared to the 25–36 months post-loss group.

### 3.4. Anxiety

Regarding self-reported symptoms of anxiety, results show that the mean scores of anxiety symptoms are the highest in the group whose loss happened 7–12 months before participation, and the effect of time post-loss on anxiety scores was not significant [*F*(6, 866) = 2.02, *p* = 0.06]. The effect size was small, η^2^ = 0.014 (Table 3).

**Figure 2:**
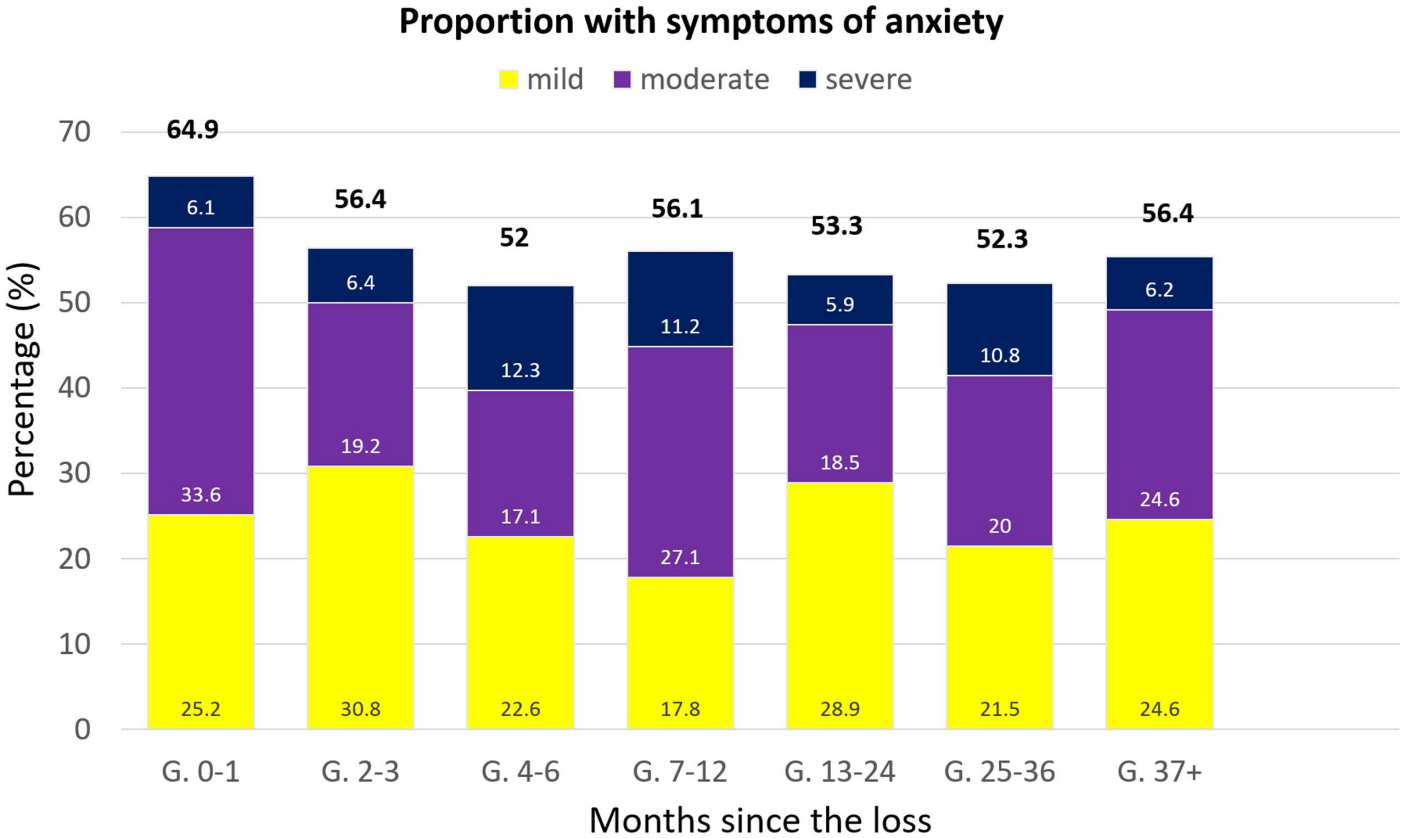
Bar chart representing the percentage of individuals showing above-threshold symptoms of anxiety, categorized according to the time passed between the loss and their participation in the study.

To compare the percentage of individuals showing the presence of anxiety symptoms in each group, we used the following cut-off: mild (8–10), moderate (11–14), and severe (15–21) (47) (Figure 2). A chi-square (χ^2^) analysis of the proportions of mild-to-severe symptoms of anxiety across groups showed no significant differences, χ^2^ (18, *N* = 873) = 27.36, *p* = 0.07. The effect size was small-to-medium, with 𝝓 = 0.10. Greater proportions of clinical symptoms of anxiety were observed in the 0–1-month group, being the majority moderate symptoms. After a decline in frequency in the 2–3 months post-loss group, there were no greater changes in numbers and, with the exception of mostly moderate symptoms in the 7–12 and 37+ group, mild symptoms were more common, and cases of severe anxiety were very few across the groups (Figure 2).

### 3.5. Depression

Regarding depression symptomatology, the mean scores are the highest in the 0–1 months post-loss group (Table 3). There was a significant effect of time post-loss on depression scores [*F*(6, 866) = 2.80, *p* = 0.01]. The effect size was small, η^2^ = 0.019. *Post-hoc* comparisons showed that in comparison to Group 0–1, the mean score is significantly lower in Group 13–24, only.

**Figure 3:**
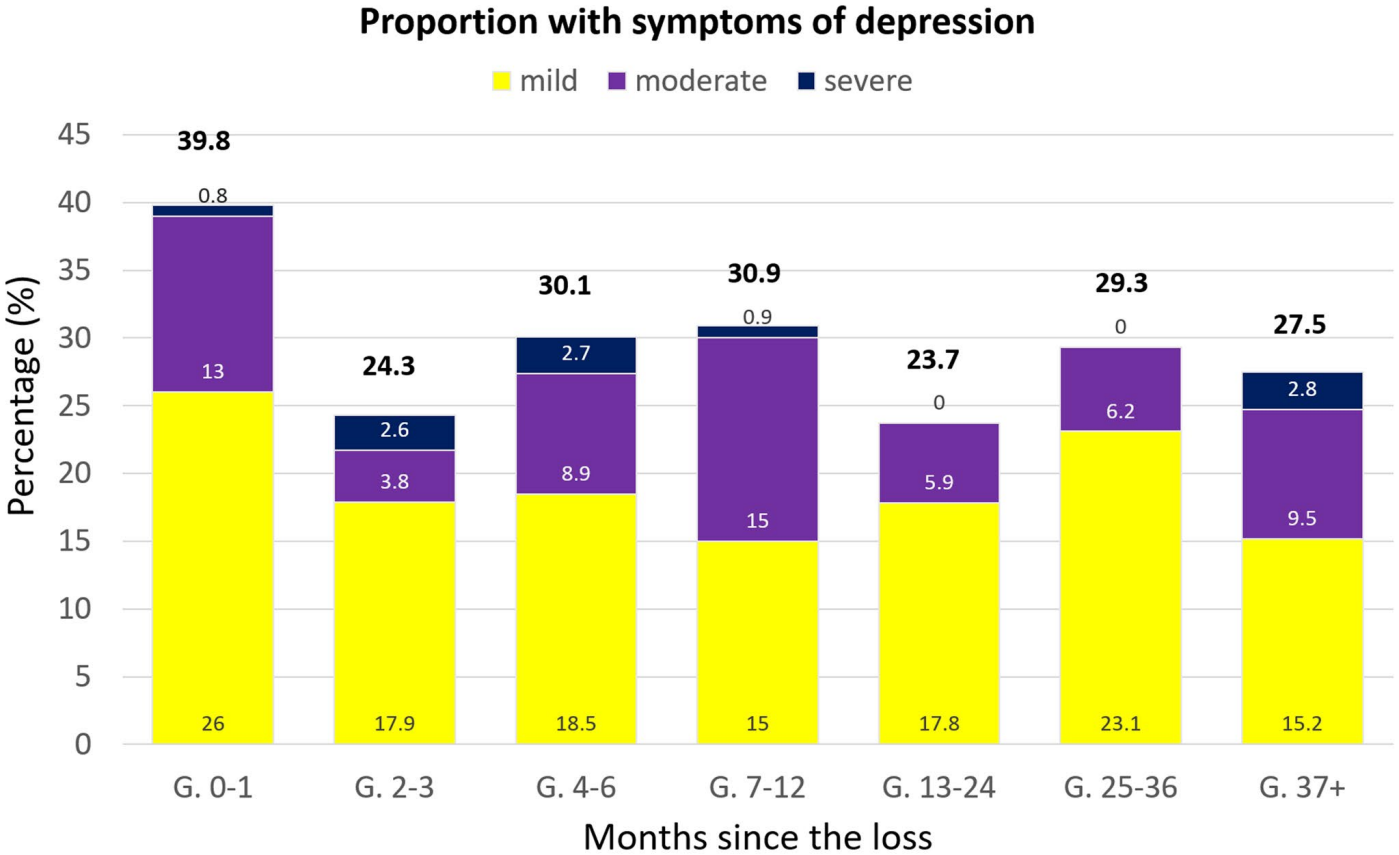
Bar chart representing the percentage of individuals showing above-threshold symptoms of depression, categorized according to the time passed between the loss and their participation in the study.

A comparison of the number of our participants showing above-threshold (mild-to-moderate) depression scores in each group based on the following cut-off scores: mild (8–10), moderate (11–14), and severe (15–21) (47) (Figure 3), showed no significant differences, χ^2^ (18, *N* = 873) = 27.922, *p* = 0.06 (25% with expected count less than 5). The effect size was small-to-medium, with 𝝓 = 0.10. The highest frequency of cases was found in the 0–1 months post-loss group, most corresponding to mild cases. A drop in cases can be seen in the 2–3 months post-loss group when compared to the 0–1 months post-loss group. Nevertheless, it was followed by greater proportions and oscillation among the groups with losses older than 3 months, with the second highest proportion in the 7–12 months, and the lowest in the 13–24 months post-loss group. Most timeframes showed a majority with mild symptoms, with only the 7–12 months post-loss showing the same percentage of mild and moderate symptoms. Proportions of severe symptoms of depression were very low in general.

### 3.6. Post-traumatic stress disorder

The mean scores of self-reported symptoms of PTSD indicate that the greatest mean score corresponds to the 0–1 months post-loss time frame. The effect of time post-loss on PTSD symptoms was significant [*F*(6, 866) = 4.41, *p* < 0.001]. The effect size was small-to-medium, η^2^ = 0.030. In comparison to Group 0–1, the mean score decreased significantly after the 13–24 months mark (Table 3).

**Figure 4:**
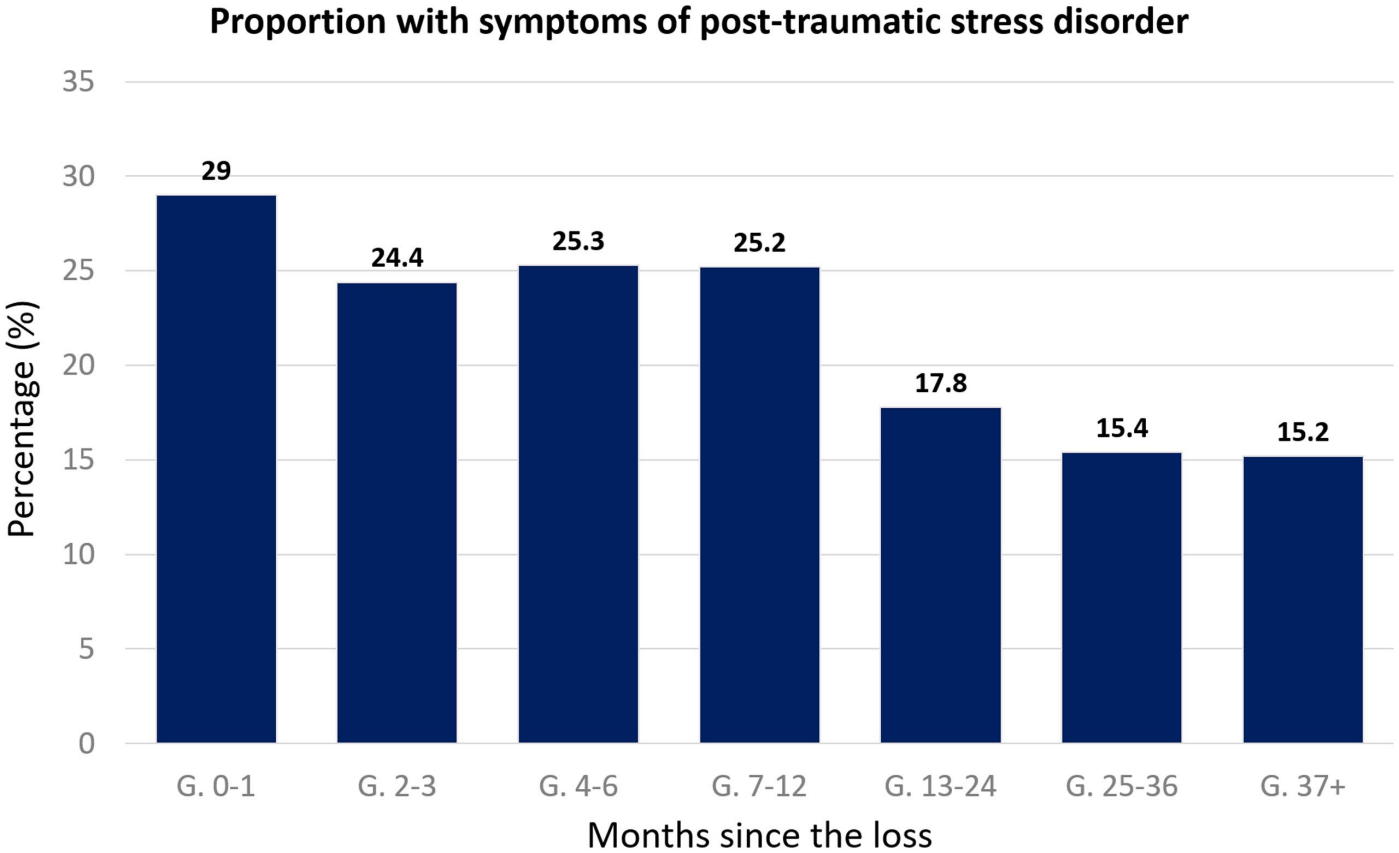
Bar chart representing the percentage of individuals showing above-threshold symptoms of post-traumatic stress disorder, categorized according to the time passed between the loss and their participation in the study.

We compared the differences in proportions of individuals with probable PTSD within the groups, and a cut-off score of 33 in the PCL-5 was used (55). A chi-square (χ^2^) analysis of the differences in frequency of Post-Traumatic Stress Disorder symptoms across groups showed significance, χ^2^(6) = 14.5, *p* = 0.02. The effect size was small-to-medium, with 𝝓=0.13. It is visible that the highest number of cases corresponds to the 0–1 months post-loss group and they gradually decreased in the following groups until the fewest cases were found in the 37+ months post-loss group (Figure 4).

## 4. Discussion

Early Pregnancy Loss can be a devastating event for some parents. Even though most surpass their grief naturally, a non-neglectable proportion of women has shown detrimental mental health responses after the event, being complicated grief, depression, anxiety, and PTSD, the most common morbidities reported around the world. This study aimed to characterize the impact this kind of loss has had in Portugal-resident women in terms of the aforementioned clinical morbidities.

Regarding perinatal grief scores, our results showed that there was a significant effect of time on these scores, with significant reductions starting at the 4–6 months mark. These results differ from those obtained by the study performed by deMontigny et al. (22), where it was concluded that perinatal grief did not vary significantly according to the time after the loss. It is important to note that deMontigny’s study, similarly to ours, was also cross-sectional and obtained data through a self-reporting online questionnaire, but they collected data from a smaller sample (231 women) and obtained slightly lower mean scores of perinatal grief for the 7–12 months, 1–2 years, and over 2 years marks in comparison (22). A closer look at the frequency of above-threshold scores of perinatal grief indicated that almost half of the population in our sample (48.1%) whose loss happened within 1 month showed potentially clinical symptoms of perinatal grief. These values are close to those obtained in a cohort study conducted in Sri Lanka (13) which assessed a sample of 137 women using the PGS, and whose results showed that up to 54% of their sample met clinical levels of perinatal grief at 6–10 weeks after EPL. The proportions of clinically relevant symptoms of perinatal grief in our sample gradually decreased, reaching their lowest point in the 25–36 months post-loss group. Nevertheless, almost a quarter (24.2%) of our sample whose loss occurred over 3 years ago showed scores above the cut-off levels. Interestingly, studies such as Krosch et al. (21) show much more concerning proportions, with 57% of their sample showing above cut-off levels of perinatal grief 4 years after the loss, a time when intense feelings of grief are expected to have resolved. On one hand, these results might suggest that a small but still important proportion of women who go through an EPL most likely had difficulties resolving their grief, still feeling deeply affected by the event even today, making follow-up evaluations essential to identify women in need of psychological support. On the other hand, this study was disseminated mostly online, which makes it susceptible to self-selection bias. This means that a proportion of women who have successfully resolved their loss or did not consider it a traumatic event might not have had interest in participating. As a result, women whose loss is still unresolved might be overrepresented in these groups whose loss happened over 3 years before.

When it comes to anxiety, our results revealed no effect of time since the loss on its scores. These results were similar to early studies carried out by Broen et al. and Nikcévic et al. which showed no significant changes in anxiety scores over time (18, 33). A more descriptive analysis of the proportions of clinical levels of anxiety in these groups indicate that, ranging from mild to severe, they remain above 50% even in the population whose loss happened 3 years before or over. Most of our population who had suffered the loss within 1 month had symptoms of moderate anxiety, while they were mostly mild in populations whose loss happened between 3 and 6 months before. Proportions of moderate-to-severe levels of anxiety symptoms at 1 and 3 months after the loss in our sample were quite similar to two studies carried out by Farren et al., common ranges being 30–40% at 1 month, and 20–26% at 3 months (24, 25). However, while the latter study by Farren et al. (25) showed a score of moderate-to-severe levels reduced to 22% at 9 months, our group whose loss happened between 7 and 12 months before showed an increase in proportions close to levels reported in the group of 0–1 months post-loss. Results obtained both in our study and in Farren et al.’s suggest that, in some cases, symptoms could spike in those months near the anniversary of the loss or when the baby could have been around a year old had the pregnancy succeeded. It is also worth to note how high these proportions are even though most women in all groups reported not having been previously diagnosed with any mental illness, a factor that has been associated to an increase in the likeliness of developing prolonged symptoms of psychological morbidities (60, 61). Even more concerning is the fact that most women in all groups reportedly never received any mental health treatment, which might explain the frequencies of above-threshold scores.

Regarding depression symptoms, our results showed a significant effect of time-after-loss in depression scores, with the only significant reduction in scores showing when comparing the 0–1 months post-loss group to the 13–24 months one. This significant reduction in depression symptoms might be related to the fact that most women in this group reported having living children, a factor that authors like deMontigny have associated to the reduction of depression scores (12, 22). However, this does not explain why this significant reduction is not visible in the other groups with older pregnancy losses, whose majority also reported having living children. Further, even though most in these groups reported having never received mental health support or treatment, over a quarter of the women whose loss happened within 7–12 months before participation reported having received mental health treatment in the past, which might have influenced this result. When analyzing the frequency of above-threshold scores of depression, there seems to be much less morbidity in comparison to anxiety, always present in less than 40% of all groups. Even though above-threshold scores of depression were found more frequently in the population whose loss happened within 1 month, it remains visible in a proportion of around 20–30% of women whose loss happened within 2 months or over 3 years before. Regardless of the time after the loss, most of these correspond to symptoms of mild depression. When it comes to the proportions of our sample showing symptoms of moderate-to-severe depression, our results are similar to a study performed by Farren et al. (24), referring specifically to the 1-month and 3 months mark. However, even though a more recent study by Farren et al. (25) showed a steady decline of moderate-to-severe levels of depression by the 9-month mark, our group whose loss happened within 7–12 months before showed a slight increase in proportions compared to 2–3 months, and even 0–1-month groups. Our results are, again, consistent with deMontigny et al.’s, who reported an increase in depression symptoms 1–2 years after the loss that disrupts the generally gradual decline (22, 25). Again, this spike in proportions might be related to the fact that a number of participants belonging to this group are likely close to the anniversary of the loss, a factor that could play part in an increase of stronger symptomatology. Altogether, our study provides evidence that symptoms of anxiety and depression could persist for 1–3 years after the event, stressing the need of periodical screening of these women’s mental condition in order to detect complicated responses to the loss and provide support if needed.

The analysis of our sample revealed a significant decrease over time in the mean scores of PTSD symptoms, with the most significant decrease visible after the 13–24 months mark. When describing the different groups in terms of the frequency of above-threshold scores of PTSD symptoms, these are in accordance with what has been reported in the literature, although none of the studies found used the same instruments we did. A study by Farren et al. (24) has reported a proportion of 28% likely to meet the criteria for moderate-to-severe PTSD at 1 month, and 39% of women meeting such criteria 3 months after EPL, showing an increase in time. Regardless, in our study, the greatest proportion of women with symptoms of PTSD was found in the group whose loss happened within 1 month, similar but lower proportions appear in the groups whose loss happened between 2 months and 1 year before, and even lower proportions in the groups whose loss happened over 1 year before show symptoms. Our results, therefore, are more similar to those reported by a more recent study by Farren et al. (25), where the percentages decreased gradually from 1 month (34%), through 3 months (26%) to 9 months after the loss (21%). Nevertheless, we cannot ignore studies like Krosch et al ’s (21), where out of a population with a mean time after the loss of 4 years, 43.9% showed symptoms of PTSD, indicating that the number of women showing relevant symptoms of PTSD years after the loss could be higher than commonly believed, possibly influenced by personal and/or environmental circumstances unique for each population. In a nutshell, despite a generally gradual decline, we cannot reject the possibility that a percentage of these women remain with high levels of PTSD long after the event and are in need of further psychological evaluation or even an intervention, making longitudinal screening of symptoms important to provide timely support for those in need and avoid severe deterioration of these women’s mental health. Overall, the results for our sample in Portugal are generally in line with those from studies that took place in other parts of the world, and in different times and contexts, with the percentages of the population showing symptoms of clinical perinatal grief, depression, anxiety, and PTSD lying within those reported by other authors, and there seems to be an effect of time in the persistence of all morbidities, except anxiety. Our results are in accordance with other studies that showed no effect of time in the prevalence of anxiety symptoms, and its greater morbidity when compared with depression.

We believe that there are some factors that could have modulated our results. Most participants in all groups indicated that the clinical cause of their loss was unknown (70.2–81.5%), a factor that has been reported to influence negatively the parents’ grieving, closure, and recovery process (18, 46). Additionally, other factors related to the event, such as the presence of social support, or the healthcare management, might have also played a part in the persistence of mental health complications in some individuals (22). deMontigny’s work showed that the women’s level of satisfaction with the health care received was significantly associated with reported symptoms of perinatal grief, anxiety, and depression (22). Therefore, this is an aspect that deserves future consideration. Factors related to the participants’ clinical history might also be worth studying. For example, it would be interesting to analyze whether having had an earlier infertility diagnosis or having recurred to MAR techniques had an influence in those women who showed above-threshold symptoms of mental health morbidities. For instance, a study by Mutiso and colleagues showed that mode of conception strongly influenced the presence of depression symptoms after miscarriage (62). It is worth highlighting that some of these losses, specifically those that happened up to 2 years before the study, happened in the context of the COVID-19 pandemic. The WHO warned on its official website at the time, more specifically its regional office for Europe, that “the main psychological impact to date is elevated rates of stress or anxiety” (63). Further, lockdown measures were taken in many countries, including Portugal, which deeply disrupted people’s routines and usual activities, to which the WHO expressed that “levels of loneliness, depression, harmful alcohol and drug use, and self-harm or suicidal behavior are also expected to rise” (64). Hence, in the context of the pandemic, not only social isolation and routine disruption might have been partially responsible for increased levels of depression and anxiety, but also some of these women might have suddenly lost family members and/or friends to the virus, situations likely to have a strong influence in the development of complicated grief or even PTSD symptoms.

This study has some limitations that need to be considered. The data were collected through an online survey. While this methodology is excellent for gathering a large sample that is geographically dispersed, and has also been used by other authors [e.g. (22)] when analyzing the effect of time, this comes from reports of different women in different moments after their losses. A longitudinal within-subjects study might have shown slightly different trends in the results. Furthermore, our data is strongly inclined to a specific population, with a majority of women in all groups being non-immigrant, white Portuguese, married, with higher education, employed, earning more than minimum wage, and resident in big cities like Lisbon and Porto. This means that women who are immigrants, from ethnic minorities, unemployed, earning a precarious wage, or living in less developed/countryside areas of Portugal are underrepresented in our sample. Additionally, many individuals who have suffered this kind of loss might have declined to take part in the study either because the event had no relevant effect on their lives, or because they might not have wanted to be reminded of such an unhappy event by the questionnaire. In fact, it is common that women with stronger symptoms of trauma avoid engaging in activities that make them confront the event of the loss (24). Hence, our results might be lacking insight into individuals belonging to the extremes of both sides of the spectrum.

The effect of Early Pregnancy Loss on women’s mental health is an important but underserved area, especially in Portugal. Our findings have highlighted that, despite not being a majority, a proportion of women still show symptoms of clinical levels of PG 6 months or over a year after the event. Furthermore, symptoms of other comorbidities such as depression, anxiety, and PTSD are also prevalent in a few cases, regardless of the time since the loss occurred. Considering this, we believe that it is essential that follow-up consultations after EPL include more than a check-up on their physical health. Rather, follow-up consultations should include screenings of PG, anxiety, depression, and PTSD to help detect any possible development of adverse mental health responses to the loss. Even though high levels of these morbidities are normal to be found within the first weeks or months after the event, they should diminish or dissipate after 6 months, so we believe the period between six-month to one-year post-loss to be ideal for screening for cases that require further attention or might benefit from counseling or psychological support.

Despite the importance of providing follow-up to the mental well-being of women who have suffered EPL, health professionals may find different limitations to providing such a service, such as lack of time, training or any follow-up protocol to follow (Catlin et al., 2017(65); Litlemore et al., 2019 (66). Some studies in countries such as Canada, the United States, and the United Kingdom, have provided evidence of the significant impact that factors like childlessness, a history of mental health illness, lack of social support, or marital satisfaction have on these women’s mental distress (22) (Barat et al., 2020) (67). Therefore, we believe that by paying special attention to the social situation and clinical history of these women, health practitioners would be able to at least prioritize mental health follow-up for women whose circumstances make them more prone to develop complicated mental health responses.

Finally, future studies should focus on the factors that may predict complicated mental health responses in Portugal. Through our study, we showed that time may not always “cure it all” and does not ensure mental well-being after a miscarriage. However, longitudinal studies on factors that might work as predictors of long-term complicated mental health responses still need to be studied in Portugal, especially because some may vary across cultures. For instance, infertility diagnosis might be a stronger predictor of declining mental health after a miscarriage in cultures where the woman’s main value is procreation and family care.

## Data availability statement

The original contributions presented in the study are included in the article/supplementary material; further inquiries can be directed to the corresponding authors.

## Ethics statement

The studies involving human participants were reviewed and approved by the Ethics Committee of the University of Madeira. The participants provided their informed consent to participate in this study.

## Author contributions

DM, AF, and MC defined and designed the study and interpreted the results. DM collected and analyzed the data. All authors contributed to the article and approved the submitted version.

## Funding

This work was supported by the Fundação para a Ciência e Tecnologia through the scholarship UI/BD/151404/2021, the AViR project (EXPL/CCI-INF/0298/2021), and NOVA LINCS (UIDB/04516/2020).

## Conflict of interest

The authors declare that the research was conducted in the absence of any commercial or financial relationships that could be construed as a potential conflict of interest.

## Publisher’s note

All claims expressed in this article are solely those of the authors and do not necessarily represent those of their affiliated organizations, or those of the publisher, the editors and the reviewers. Any product that may be evaluated in this article, or claim that may be made by its manufacturer, is not guaranteed or endorsed by the publisher.
